# Who is more likely to ignore experts' advice related to COVID-19?

**DOI:** 10.1016/j.pmedr.2021.101470

**Published:** 2021-06-26

**Authors:** Brian A. O'Shea, Michiko Ueda

**Affiliations:** a Department of Psychology, University of Amsterdam, the Netherlands; b Department of Psychology, Harvard University, USA; c Faculty of Political Science and Economics, Waseda University, Japan

**Keywords:** COVID-19, Trust, Experts, Germ Aversion, Demographics

## Abstract

Failing to adhere to COVID-19 experts’ advice could have devastating consequences for individuals and communities. Here we determine which demographic factors can impact trust in COVID-19 experts. Participants consisted of more than 1875 online volunteers, primarily from the U.S. Survey data were collected before and after the first peak of the COVID-19 outbreak in the U.S. (28th of March−15th of May 2020). We consistently find that participants with a lower perceived socio-economic status, social conservatives, individualists, and participants who are less worried about COVID-19 are significantly *more* likely to support individuals who ignore the goverment's, scientists’, medical professionals’ COVID-19 advice. Regarding race, Black participants consistently (and Hispanics to a lesser degree) were *more* likely to support individuals who ignore the three expert groups relative to Whites. All these findings generalized to weaker trust towards public policy decision experts. Asian and other racial groups’ trust was consistently lower than Whites, but primarily numerically, not statistically. Age and gender showed weak or inconsistent results respectively. We provide an enhanced understanding of the demographic factors that can result in individuals/groups ignoring COVID-19 experts. Lack of compliance could increase the transmission risks of the virus. Therefore, non-partisan campaigns that target individuals/groups who distrust COVID-19 experts will likely reduce COVID-19 related deaths. Increasing expert representatives’ racial diversity may also increase trust among racial minorities.

## 1 Introduction

Trust in science and the government has been extremely critical during the COVID-19 pandemic (Liang et al., 2020). Due to the slow vaccine rollout, engagement in preventative behaviors is still crucial for containing the spread of the novel coronavirus (Howard et al., 2020). Many governments have introduced various recommendations and restrictions based on the best available scientific evidence, ranging from the use of face masks to strict movement restrictions. As some of these measures entail severe inconvenience for daily activities and an intrusion on individual freedom, achieving a high compliance rate requires that people trust what they are being told; Trust in scientific evidence, the medical community, government actions and policy experts are all crucial for achieving a high degree of adherence to public health instructions.

However, a substantial number of people do not trust the government, science in general, or the medical community. For example, trust in the U.S. government is near historic lows at 17% (Pew Research Center. Public Trust in Government:, 2019). Moreover, the percentage of U.S. citizens who have a great deal of confidence in the scientific community and medicine has remained stable for many years, and in 2018, it was reported to be at 44% and 37%, respectively (Funk and Kennedy, 2020). However, recently, trust in medical scientists has increased in the U.S. but mainly among liberals (Funk et al., 2020a), likely impacted by the politicization of COVID-19 (Thacker, 2020). In addition, skeptical attitudes towards science are known to be prevalent among some segments of the population, including those who are politically conservative (Gauchat, 2012, Blank and Shaw, 2015), religious (Cacciatore et al., 2018, Miller, 2004), Black Americans (Funk et al., 2020b; Corbie-Smith et al., 1999), and those with a lower socio-economic status (Dunlop et al., 2000).

This paper is an exploratory investigation of who is more likely to support those who ignore or dismiss experts’ health advice and government recommendations by analyzing data from a survey of individuals conducted in the midst of the COVID-19 outbreak. We will also address which individuals/groups are more likely to trust experts than ordinary people regarding policy decisions. Although we made no specific hypotheses prior to analyzing the data, we feel that an intergroup framework, specifically the conflict (political, economic, power) between dominant/high-status groups versus subordinate/lower-status groups, might be useful to contextualize our findings.

In short, humans are attuned to identifying social groups based on numerous dimensions such as race, social status, gender, religion etc. (Tajfel and Turner, 2001, Turner et al., 1987). Moreover, in-group preference (and/or outgroup derogation) is a common occurrence (Greenwald and Pettigrew, 2014). Humans can even form groups based on trivial criteria (e.g., preference for paintings or the toss of a coin) and show clear preferences towards these arbitrary groups (Diehl, 1990). Concordantly, individuals are more likely to trust and adhere to guidance/recommendations from those they perceive to represent their in-group (Williams, 2001). For one to become an expert in science, medicine or government policy, many years of education are needed, and the high cost of education in countries like the US, may negatively impact social mobility of very capable candidates from ‘low-status groups’ (Johnson and Peifer, 2017, Wilkinson and Pickett, 2009). Therefore, if those who reach the highest levels of institutions are disproportionately wealthier, whiter, more liberal from elite schools, individuals/groups who do not see themselves as being represented by those giving COVID-19 recommendations may be less likely to trust or adhere to the recommendations.

There is emerging evidence that certain groups are less likely to engage in COVID-19 preventive behaviors, including those with lower socio-economic status (Papageorge et al., 2021), and U.S. Republicans (conservatives) (Partisanship, 2020). However, we have little understanding of who is more likely to support those who ignore COVID-19 experts’ advice/recommendations (a proxy for participants’ likelihood of distrust) and how these individuals vary by important demographic factors or individual differences. Here we gather a wide variety of demographic variables, including psychological scales relevant to infectious diseases (perceived vulnerability to disease) and collective behaviors (individualism versus collectivism). These scales and conceptual frameworks are often used to determine variation in individuals’ hypervigilance response to infectious diseases (see behavioral immune system research (Schaller and Park, 2011)) or how regions with a higher prevalence of infectious diseases are related to collectivistic worldviews (see parasite stress theory (Thornhill and Fincher, 2014)). Here we aim to illuminate crucial individual and group differences that may contribute to the rejection of COVID-19 expert advice.

## 2 Methods

### 2.1 Participants and procedure

Harvard University granted IRB approval for this study. The study link appeared on the front page of the Project Implicit website (implicit.harvard.edu) between the 28th of March−15th of May 2020. The link was called “Project Implicit COVID-19 Task” and the description below the link was “COVID-19 BIAT. This BIAT requires the ability to recognize photos of landmarks from the UK, US, China, and Italy. It will test how you associate COVID-19 with these countries/people”. Moreover, the informed consent detailed how the study is directly related to COVID-19 (e.g., “This study will examine your implicit and explicit attitude towards COVID-19”). Therefore, this exposure/priming ensures participants were providing responses in a COVID-19 context and not just providing general sentiments. After selecting a button to indicate agreement with the informed consent, participants completed in random order, demographic questions, and various questionnaires, including measures of implicit and explicit attitudes related to COVID-19. Only the variables that are relevant to our research question were analyzed. The full experiment can be viewed by copying the following link into your browser. https://app-prod-03.implicit.harvard.edu/implicit/Launch?study=/user/oshea/featured_biat//featured_biat.notouch.expt.xml

Informed consent was agreed upon by 3991 volunteers, and 2332 participants fully completed the study. Due to missing responses, for each analysis, we had between 1,875 to 1,880 participants with complete data. Approximately, 34% of the sample were male and 27% resided outside of the U.S., with the majority coming from the U.K., Australia and Canada. Over 91% of participants residing in the U.S. were U.S. citizens, while less than 6% residing outside the U.S. were U.S. citizens. The mean age of the sample was 31 (*SD* ≈ 13.5). *The* majority of the sample were White (≈ 59%), and hence this group was used as the race reference category in the analysis. Approximately, 14% were Asian, 7% were Black, 12% were Hispanic, and 8% were comprised of other racial groups (primarily mixed race).

## 3 Materials

### 3.1 Dependent Responses.

The questions posed in the context of a COVID-19 study were: “How warm or cold do you feel towards those who (1) ignore the government's advice (reverse coded), (2) follow scientists' advice and (3) ignore doctors'/medical professionals' advice” (reverse coded). Response options ranged from Extremely cold/negative (1) to Extremely warm/positive (10). We had various other feeling thermometer questions that always targeted a particular group (e.g., Chinese, White People, Americans) and therefore, the above questions were posed in a similar structure to facilitate participants to respond quickly and easily. Although it may be possible participant's feelings towards others who ignore experts’ advice diverges from their personal feelings towards these experts, we feel this is unlikely. Therefore, we believe the three dependent variables are also likely capturing an individual’s tendency to ignore experts’ advice. Importantly, we also posed the following question which directly captures participant’s personal trust towards experts and has been previously used in the literature (Motta, 2018, Oliver and Rahn, 2016): “When it comes to public policy decisions, whom do you tend to trust more: ordinary people or experts?” 5 response options were available, ranging from (1) “Trust ordinary people much more” to (5) “Trust experts much more”.

### 3.2 Independent variables.

The question “Please indicate the country of your residence” was used to determine a participant’s country (0 = not the U.S. and 1 = the U.S.). Age acted as a continuous variable (only 18 or older included), while gender was dummy coded (0 = female and 1 = male). Two questions were used to determine a participant’s ethnic/racial group. (A) What is your ethnicity? and (B) Please select the categories that comprise your race? The first question had three response options: (1) Hispanic or Latino, (2) Not Hispanic or Latino, (3) Unknown. A participant was classified as Hispanic (0 = White, 1 = Hispanic) if they selected the first response options, regardless of how they responded to the second question (B). A participant was classified as Asian if they selected ‘East Asian’, ‘South Asian’ or both these response options (0 = White, 1 = Asian). By selecting only ‘Black or African American’ or ‘White’ a participant was classified as Black (0 = White, 1 = Black) or White respectively (White acted as the reference category throughout). All other response options were classified as Other Race (0 = White, 1 = Other Race).

To determine education attainment, we asked: “Please indicate the highest level of education that you have completed” (1 = elementary/primary school to 10 = advanced degrees such as Ph.D.). Perceived socio-economic status was measured by the following statement appearing next to an image of a ladder. “Think of this ladder as representing where people stand in your country: At the top of the ladder are those who are the best off—those who have the most money, the most education, and the most respected jobs. At the bottom are the people who are the worst off—who have the least money, least education, and least respected jobs or no jobs. The higher up you are on this ladder, the closer you are to the people at the very top; the lower you are, the closer you are to the people at the very bottom. (SES; 1 = low SES to 10 = high SES). “How religious do you consider yourself to be?” was our question to measure religious belief (1 = not at all religious to 4 = strongly religious), and worry of COVID-19 was measured using the question: “How worried are you personally about Coronavirus?” (1 = Not worried at all to 4 = Very Worried).

“Please indicate your political identity on social issues (e.g., abortion, gun control, gay rights)” was used to determine social issues ideology, while “Please indicate your political identity on economic issues (e.g., taxation, government spending)” was used for assessing economic issues ideology (1 = strongly liberal to 7 = strongly conservative). Individualism was measured using the cultural cognition worldview scale (Kahan, 2008), and the two subscales from the perceived vulnerability to disease scale (Duncan et al., 2009) were used to estimate an individual’s infectability concern and their germ aversion tendencies.

### 3.3 Analysis

SPSS ordinal logistic regression was used to predict the four dependent variables. Each model included the above 16 independent variables. The data, full analysis, and further demographic information are available on Open Science Framework (https://osf.io/jwuz3/?view_only=c7b0d87b1bbe44f6ab97602e0e0a4c61).

## 4 Results

Throughout we will present in brackets the relative percentage difference of the predictor on the dependent variable, based on the odds ratio effects size estimates. As shown in Table 1, social conservatives (OR = 0.90–0.75; therefore the relative percentage difference is 10–25%,), individualists (20–26%), those with lower perceived social status (OR = 1.05–1.10; therefore the relative percentage difference is 5–10%) and lower COVID-19 worries (31–70%), were consistently and significantly more likely to support those who ignore (distrust) the government's, scientists’, and medical professionals/doctors’ (experts) advice in the COVID-19 context. Regarding racial differences, non-Whites were significantly more likely to support those who ignore the COVID-19 experts (see supplemental Table 1). However, the large and racially diverse sample size allowed us to perform a more nuanced analysis. The non-White finding above was primarily driven by the Black participants in the sample. Black participants showed significantly more support towards those who ignored or distrusted experts (25–64%) on the four dependent variables relative to White participants. Similar but weaker trends were shown for Hispanics (22–33%). Although Asians, and “Other Race” showed numerically more support towards those who ignored or distrusted experts than Whites, these effects never reached statistical significance, except for Asian showing more trust towards ordinary people when it comes to policy decision (28%; see Table 1).

**Table 1:** Summary of the Ordinal Logistic Regression Results.

	Government (*N* = 1,875)			Scientists (*N* = 1,877)			Medical Professionals (*N* = 1,879)			Experts (versus ordinary people) (*N* = 1,880)		
*Predictor*	*b*	*SE b*	*OR (95% CI)*	*b*	*SE b*	*OR (95% CI)*	*b*	*SE b*	*OR (95% CI)*	*b*	*SE b*	*OR (95% CI)*
Country	−0.45	0.10	0.64 (0.52, 0.78)***	0.12	0.10	1.13 (0.92, 1.38)	−0.21	0.10	0.81 (0.66, 099)*	0.14	0.12	1.14 (0.90, 1.45)
Age	−0.01	0.00	0.99 (0.99, 1.00)	0.00	0.00	1.00 (0.99, 1.01)	−0.01	0.00	0.99 (0.98, 1.00)**	0.01	0.00	1.01 (1.00, 1.02)*
Gender	−0.27	0.09	0.76 (0.64, 0.91)**	−0.03	0.09	0.97 (0.81, 1.16)	−0.09	0.09	0.92 (0.77, 1.09)	0.37	0.11	1.44 (1.17, 1.78)***
Asian	−0.13	0.13	0.88 (0.69, 1.12)	−0.18	0.12	0.84 (0.66, 1.07)	−0.23	0.13	0.80 (0.62, 1.02)†	−0.33	0.14	0.72 (0.54, 0.95)*
**Black**	−1.03	0.19	0.36 (0.25, 0.52)***	−0.86	0.18	0.42 (0.29, 0.60)***	−0.59	0.18	0.55 (0.39, 0.79)***	−1.03	0.20	0.36 (0.24, 0.53)***
Hispanic	−0.25	0.14	0.78 (0.60, 1.02)†	−0.40	0.14	0.67 (0.51, 0.87)**	−0.25	0.14	0.78 (0.60, 1.02)†	−0.31	0.15	0.73 (0.55, 0.98)*
Other Race	−0.17	0.16	0.84 (0.62, 1.15)	−0.30	0.16	0.74 (0.54, 1.01)†	−0.18	0.16	0.84 (0.62, 1.14)†	−0.25	0.18	0.78 (0.54, 1.11)†
Education	0.00	0.02	1.00 (0.95, 1.05)	0.01	0.03	1.01 (0.96, 1.07)	0.01	0.03	1.01 (0.96, 1.06)	0.05	0.03	1.05 (0.99, 1.11)
**Social Status**	0.05	0.03	1.05 (0.99, 1.11)†	0.07	0.03	1.07 (1.01, 1.13)*	0.06	0.03	1.06 (1.00, 1.12)*	0.10	0.03	1.10 (103, 1.17)**
**Social Ideology**	−0.10	0.04	0.90 (0.84, 0.97)**	−0.24	0.04	0.79 (0.73, 0.84)***	−0.18	0.04	0.84 (0.78, 0.90)***	−0.28	0.04	0.75 (0.70, 0.81)***
Economic Ideology	0.01	0.03	1.01 (0.95, 1.08)	−0.01	0.03	0.99 (0.93, 1.06)	0.03	0.03	1.03 (0.97, 1.10)	0.05	0.04	1.05 (0.97, 1.13)
Religious Belief	−0.01	0.05	0.99 (0.90, 1.09)	−0.07	0.05	0.93 (0.85, 1.02)	−0.03	0.05	0.97 (0.88, 1.07)	−0.07	0.06	0.93 (0.83, 1.04)
**Individualism**	−0.30	0.03	0.74 (0.69, 0.79)***	−0.15	0.03	0.86 (0.81, 0.92)***	−0.21	0.03	0.81 (0.76, 0.87)***	−0.23	0.04	0.80 (0.74, 0.86)***
**COVID**−**19 Worry**	0.53	0.06	1.69 (1.50, 1.91)***	0.41	0.06	1.51 (1.34, 1.71)***	0.41	0.06	1.51 (1.33, 1.70)***	0.26	0.07	1.30 (1.13, 1.49)***
Infectability Concern	−0.02	0.04	0.98 (0.91, 1.05)	0.04	0.04	1.04 (0.96, 1.12)	−0.01	0.04	0.99 (0.92, 1.07)	−0.03	0.04	0.97 (0.89, 1.07)
Germ Aversion	0.25	0.04	1.28 (1.18, 1.40)***	0.12	0.04	1.13 (1.03, 1.23)**	0.34	0.05	1.41 (1.29, 1.54)***	0.05	0.05	1.05 (0.95, 1.16)

*Note:* For the dependent variables, higher scores indicate *greater* trust (less likely to support ignoring). For the independent variables, *higher* values on each variable indicate U.S. residents, older, Asian, Black, Hispanic, Other Race (White was the reference category for the four groups), more education, higher social status, more socially conservative, more economically conservative, more religious, greater individualistic beliefs, more COVID-19 worry, greater infectability concern, and germ aversions. Predictors in bold and those underlined indicate a significant or partially significant pattern respectively. †p < .10, *p < .05, **p < .01, ***p < .001**.**

Individuals who expressed weaker germ aversion tendencies were more likely to support those who ignore the government's, scientists’, medical professionals'/doctors’ advice (13–41%), but germ aversion was unrelated to trust towards experts versus ordinary people regarding policy decisions. Moreover, U.S. residents were significantly more likely than residents of other nations in the sample to support those who ignore the government and medical professionals’ advice (19–36%), but no difference was observed for scientists' advice or trust towards policy experts. Males were significantly more likely than females to support those who ignore the government's advice (24%), while males showed more trust towards policy experts (44%). Older people were significantly more likely to show support towards those who ignored medical professionals (1%), yet they showed more trust towards policy experts (1%). All other relationships were not significant, including educational attainment, religious belief, economic ideology, and infectability concern, throughout the four dependent variables (see Table 1). Of note, the odds ratios generally indicated a small effect size. Yet we expect these findings to be meaningful for society regarding managing the pandemic due to the highly contagious nature of COVID-19 and how quickly the cases/deaths can grow exponentially (Christakis, 2020), especially if individuals/groups don’t follow COVID-19 preventative behaviors.

## 5 Discussion

Understanding which individuals/groups who are more likely to support those who ignore or distrust experts in a pandemic context should be beneficial for developing preventative public health strategies, to garner trust in order to mitigate the spread of the disease (see separate section below for more details). Here we determine the demographic factors and novel individual difference variables that relate to trust towards (or less likely to support those that ignore) experts in the COVID-19 context. We consistently find that relative to White participants, only Black participants – among Asian, Hispanics and “Other Race – consistently showed reduced trust towards COVID-19 experts. Additionally, those with a lower perceived social status relates to reduced trust towards COVID-19 experts. Like other diseases in the U.S., COVID-19 cases are disproportionately impacting racial minorities of lower socio-economic status (CDC, 2020; Dyer, 2020). Importantly, there are various systemic structural reasons for these disparities (e.g., discrimination, healthcare access, utilization, occupation, wealth gap, housing) (CDC, 2020), which could negatively impact trust towards those at the top of health, educational and governance systems (Skloot, 2010).

Social conservatism and individualism are related to distrust and support towards those who ignore experts, but economic conservatism is unrelated. The politicization of COVID-19 might explain this divergence where former U.S. President Trump consistently promoted conservative social issues, but he was less consistent regarding conservative economic issues. This finding is consistent with the social primacy hypothesis where ideological worldview conflicts are experienced more strongly along the social rather than economic political dimensions (Crawford, 2014). Finally, while an individual’s *perception* of their perceived susceptibility of contracting diseases (infectability concern) is unrelated to distrust, those with a reduced personally worry of COVID-19, and lower germ aversion *behavioral* tendencies (e.g., higher willingness to share a bottle of water) are more likely to support those who ignore COVID-19 experts. Recent evidence from a U.S. sample has shown that germ aversion is positively related to conservatism, while infectability concern and personal worry of COVID-19 are negatively related to conservatism (O’Shea et al., 2021). The impact of conservative media outlets and former U.S. President Trump were offered as potential factors accounting for these relationships, which are also likely impacting the findings reported here (Motta et al., 2020).

The handling of the COVID-19 pandemic by the Trump administration is likely contributing to U.S. residents greater distrust towards the government (Rutledge, 2020), accelerating the negative perceptions prior to the pandemic (Pew Research Center. Public Trust in Government:, 2019). Testing the impact that the Biden administration (current U.S. President) has in fostering trust in relation to handling of the pandemic and vaccine rollout will be important future work. Among the elderly and U.S. residents, their greater support for those who ignore medical professionals/doctors may be due to the extreme cost of healthcare in the U.S. relative to other high-income countries (Papanicolas et al., 2018). Determining the cross-national factors that contribute to greater support towards national healthcare systems and doctors is important future research and will likely increase adherence to medical professional recommendations, especially during a pandemic.

### 5.1 Limitations

A clear limitation of this study is the fact that the sample was not representative. Project Implicit generally attracts younger participants (online nature), more females, liberals and those with more education than the general population (Hehman et al., 2019). Additionally, only 33 gender non-binary participants were in the whole dataset and this small sample size forced us to exclude these participants and make gender a dummy variable. Therefore, any conclusions drawn from these data of online volunteers may not generalize to the population at large. Yet, Project Implicit's explicit biases show similar trends over time to nationally representative polls (Charlesworth and Banaji, 2019) and various studies have shown that results from Project Implicit samples are associated with meaningful regional outcomes (Giasson and Chopik, 2020, O’Shea et al., 2020). Importantly, we feel the varied/novel demographic questions and scales used, offer new and interesting perspectives for how individuals/groups differ relatively to one another in the context of trust and supporting the adherence to expert authorities’ recommendations during a pandemic. Crucially, future research would benefit from testing the generalizability of these findings using representative samples within and across countries.

Further limitations include (1) the COVID-19 personal worry single item question has limited ability at identifying what concerns it is capturing (e.g., effect on the country, family/friends, economic prospects, their personal health etc.), (2) using proxies for trust towards experts (i.e., supporting those who ignore experts), (3) only identifying demographic differences in attitudes toward experts, without any attempt to ascertain the causes and contributors of those differences and (4) the study’s correlational cross-sectional nature. The data were gathered at the start of the COVID-19 pandemic when lockdown measures were put in place and uncertainty was high. This data collection period likely provides unique insights into how individual/groups responded to COVID-19 experts. However, it is possible the results may change over the course of the pandemic, for example, when COVID-19 cases/deaths spiked and dipped or when the vaccine was approved and started being rolled out. It would be useful to test whether these results are also shown when society is not in a pandemic.

### 5.2 Public health implications

Only until the COVID-19 vaccine is rolled out on a mass scale can people relax the behavioral mitigations strategies recommended by COVID-19 experts. Consequently, it is beneficial for public health officials to understand who is most likely to ignore the COVID-19 (and future pandemic) preventative behavioral recommendations. This knowledge will help them to target the individuals and groups most likely to ignore or show reduced trust towards COVID-19 experts’ advice, through non-partisan public health campaigns, in an effort to change health behaviors. These targeted campaigns will likely mitigate the spread of the virus and COVID-19 related deaths, including other transmissible diseases. Moreover, increasing the diversity of expert authority representatives may also garner greater trust among individuals/groups that can identify with a representative. Based on our results, increased diversity in race, ideology and social class seems like an obvious strategy to increase trust. However, it is imperative that these representatives present a unified message to increase the likelihood of garnering similar trust levels across groups.

## 6 Conclusion

Conflict (symbolic or otherwise) between dominant groups, who are often in positions of power, versus less dominant groups, may impact trust towards expert authorities. Here we show that social conservatives, individualists, and those with lower perceived socio-economic status, including COVID-19 worries are more likely to show support towards those who ignore various experts in the COVID-19 context. Racial minorities, especially Black respondents also showed reduced support and trust towards experts. Public health campaigns targeting these groups and increasing diversity in positions of authority were offered as potential strategies to mitigate distrust towards experts.

## CRediT authorship contribution statement

**Brian A. O’Shea:** Conceptualization, Data curation, Formal analysis, Funding acquisition, Writing - original draft, Writing - review & editing. **Michiko Ueda:** Writing - original draft, Writing - review & editing.

## Declaration of Competing Interest

The authors declare that they have no known competing financial interests or personal relationships that could have appeared to influence the work reported in this paper.
