# Supplementary material for: Who is more likely to ignore experts' advice related to COVID-19?
Source: Prev Med Rep. 2021 Jun 26;23:101470. doi: 10.1016/j.pmedr.2021.101470 (PMC8261004; doi:10.1016/j.pmedr.2021.101470)
Supplement: Supplementary data 1 [file mmc1.docx]

**Title:** Who is more likely to ignore experts' advice related to COVID-19?

**Supplemental Materials**

Brian A. O’Shea^1,2^*, Michiko Ueda^3^.

1. Department of Psychology, University of Amsterdam, the Netherlands
2. Department of Psychology, Harvard University, USA
3. Faculty of Political Science and Economics, Waseda University, Japan

*Correspondence concerning this article should be addressed to Brian Agh. O’Shea, Department of Psychology, University of Amsterdam, Roetersstraat 15, 1018 WB Amsterdam, The Netherlands
E-mail: [b.a.oshea@uva.nl](mailto:b.a.oshea@uva.nlu).

|  | Government (*N* = 1,875) | | | Scientists (*N* = 1,877) | | | Medical Professionals (*N* = 1,879) | | | Experts (versus ordinary people) (*N* = 1,880) | | |
| --- | --- | --- | --- | --- | --- | --- | --- | --- | --- | --- | --- | --- |
| *Predictor* | *b* | *SE b* | *OR (95% CI)* | *b* | *SE b* | *OR (95% CI)* | *b* | *SE b* | *OR (95% CI)* | *b* | *SE b* | *OR (95% CI)* |
| Country | -0.50 | 0.10 | 0.61 (0.50, 0.74)*** | 0.08 | 0.10 | 1.08 (0.88, 1.31) | -0.23 | 0.10 | 0.79 (0.65, 0.97)* | 0.10 | 0.12 | 1.10 (0.87, 1.40) |
| Age | -0.01 | 0.00 | 0.99 (0.99, 1.00) | 0.00 | 0.00 | 1.00 (0.99, 1.01) | -0.01 | 0.00 | 0.99 (0.98, 1.00)** | 0.01 | 0.00 | 1.01 (1.00, 1.02)† |
| Gender | -0.26 | 0.09 | 0.77 (0.65, 0.92)** | -0.03 | 0.09 | 0.97 (0.81, 1.16) | -0.08 | 0.09 | 0.92 (0.77, 1.10) | 0.38 | 0.11 | 1.46 (1.18, 1.80)*** |
| **Race** | 0.33 | 0.09 | 1.39 (1.16, 1.67)*** | 0.31 | 0.09 | 1.37 (1.15, 1.64)*** | 0.30 | 0.09 | 1.35 (1.13, 1.62)*** | 0.40 | 0.10 | 1.49 (1.21, 1.82)*** |
| Education | 0.00 | 0.02 | 1.00 (0.96, 1.04) | 0.00 | 0.02 | 1.00 (0.96, 1.04) | 0.00 | 0.02 | 1.01 (0.96, 1.04) | 0.05 | 0.03 | 1.06 (0.99, 1.12)† |
| **Social Status** | 0.06 | 0.03 | 1.06 (1.01, 1.12)* | 0.08 | 0.03 | 1.09 (1.03, 1.15)** | 0.06 | 0.03 | 1.06 (1.01, 1.13)* | 0.10 | 0.03 | 1.11 (1.04, 1.18)** |
| **Social Ideology** | -0.09 | 0.04 | 0.91 (0.85, 0.98)** | -0.24 | 0.04 | 0.79 (0.73, 0.84)*** | -0.18 | 0.04 | 0.84 (0.78, 0.90)*** | -0.28 | 0.04 | 0.76 (0.70, 0.82)*** |
| Economic Ideology | 0.02 | 0.03 | 1.02 (0.95, 1.08) | 0.00 | 0.03 | 1.00 (0.93, 1.07) | 0.03 | 0.03 | 1.03 (0.97, 1.10) | 0.06 | 0.04 | 1.06 (0.98, 1.14) |
| Religious Belief | -0.03 | 0.05 | 0.97 (0.88, 1.06) | -0.09 | 0.05 | 0.91 (0.83, 1.00) | -0.04 | 0.05 | 0.96 (0.88, 1.06) | -0.09 | 0.06 | 0.91 (0.82, 1.01)† |
| **Individualism** | -0.31 | 0.03 | 0.73 (0.69, 0.78)*** | -0.115 | 0.03 | 0.86 (0.81, 0.92)*** | -0.21 | 0.03 | 0.81 (0.76, 0.86)*** | -0.23 | 0.04 | 0.79 (0.74, 0.85)*** |
| **COVID-19 Worry** | 0.53 | 0.06 | 1.70 (1.51, 1.92)*** | 0.41 | 0.06 | 1.51 (1.34, 1.71)*** | 0.41 | 0.06 | 1.51 (1.34, 1.70)*** | 0.26 | 0.07 | 1.29 (1.13, 1.48)*** |
| Infectability Concern | 0.00 | 0.04 | 1.00 (0.93, 1.07) | 0.06 | 0.04 | 1.06 (0.98, 1.14) | 0.00 | 0.04 | 1.00 (0.93, 1.08) | -0.01 | 0.04 | 0.99 (0.91, 1.08) |
| **Germ Aversion** | 0.23 | 0.04 | 1.26 (1.16, 1.37)*** | 0.10 | 0.04 | 1.11 (1.01, 1.21)* | 0.33 | 0.04 | 1.40 (1.28, 1.53)*** | 0.03 | 0.05 | 1.03 (0.94, 1.14) |

Supplemental Table 1. Summary of the Ordinal Logistic Regression Results

*Note:* For the dependent variables, higher scores indicate *greater* trust (less likely to support ignoring). For the independent variables, *higher* values on each variable indicate U.S. residents, older, male, White, more education, higher social status, more socially conservative, more economically conservative, stronger religious belief, greater individualistic beliefs, more COVID-19 worry, greater infectability concern, and germ aversions. Predictors in bold indicate a consistent pattern. †p<.10, **p*<.05, ***p*<.01, ****p*<.001
